# A curated compendium of monocyte transcriptome datasets of relevance to human monocyte immunobiology research

**DOI:** 10.12688/f1000research.8182.2

**Published:** 2016-04-25

**Authors:** Darawan Rinchai, Sabri Boughorbel, Scott Presnell, Charlie Quinn, Damien Chaussabel

**Affiliations:** 1 Systems Biology Department, Sidra Medical and Research Center, Doha, Qatar; 2 Biomedical Informatics Division, Sidra Medical and Research Center, Doha, Qatar; 3 Benaroya Research Institute at Virginia Mason, Seattle, USA

**Keywords:** Monocyte, Transcriptomics, Gene Expression Browser, Immunology, Bioinformatics

## Abstract

Systems-scale profiling approaches have become widely used in translational research settings. The resulting accumulation of large-scale datasets in public repositories represents a critical opportunity to promote insight and foster knowledge discovery. However, resources that can serve as an interface between biomedical researchers and such vast and heterogeneous dataset collections are needed in order to fulfill this potential. Recently, we have developed an interactive data browsing and visualization web application, the Gene Expression Browser (GXB). This tool can be used to overlay deep molecular phenotyping data with rich contextual information about analytes, samples and studies along with ancillary clinical or immunological profiling data. In this note, we describe a curated compendium of 93 public datasets generated in the context of human monocyte immunological studies, representing a total of 4,516 transcriptome profiles. Datasets were uploaded to an instance of GXB along with study description and sample annotations. Study samples were arranged in different groups. Ranked gene lists were generated based on relevant group comparisons. This resource is publicly available online at http://monocyte.gxbsidra.org/dm3/landing.gsp.

## Introduction

Platforms such as microarrays and, more recently, next generation sequencing have been leveraged to generate molecular profiles at the scale of entire systems. The global perspective gained using such approaches is potentially transformative. Transcriptome profiling enabled for instance the characterization of molecular perturbations that occur in the context of a wide range disease processes ^1– 10^. This in turn has provided opportunities for the discovery of biomarkers and for the development of novel therapeutic modalities ^3, 11– 13^. More recently such systems-scale profiling of the blood transcriptome has also been used to monitor response to vaccines or therapeutic drugs ^14– 19^. The democratization of these approaches has led to proliferation of data in public repositories: over 1.7 million individual transcriptome profiles from more than 65,000 studies have been deposited to date in the NCBI Gene Expression Omnibus (GEO), a public repository of transcriptome profiles.

Taken together this vast body of “collective data” holds the promise of accelerating the pace of biomedical discovery by creating countless opportunities for identifying and filling critical knowledge gaps. Building tools that provide biomedical researchers with the ability to seamlessly interact with collections of datasets along with rich contextual information is essential in promoting insight and enabling knowledge discovery. To address this need we have developed an interactive data browsing and visualization web application, the Gene Expression Browser (GXB).

GXB was described in a recent publication and is available as open source software on GitHub ^20^. This tool constitutes a simple interface for the browsing and interactive visualization of large volumes of heterogeneous data. Users can easily customize data plots by adding multiple layers of information, modifying the order of samples, and generating links that capture these settings, which can be inserted in email communications or in publications. Accessing the tool via these links also provides access to rich contextual information that is essential for data interpretation. This includes access to gene information and relevant literature, study design information, detailed sample information as well as ancillary data ^20^.

In recent years, a large number of transcriptional studies have been conducted aiming at the characterization and functional classification of monocytes in health and disease. Monocytes are a population of immune cells found in the blood, bone marrow, and spleen. They constitute ~10% of the total circulating blood leukocytes in humans. They can remain in the blood circulation for up to 1–2 days, after which time, if they have not been recruited to a tissue, they die and are removed. They are considered the systemic reservoir of myeloid precursors for renewal of tissue macrophages and dendritic cells. Monocytes play a key role during immune response as professional phagocytes ^21, 22^, and producers of immune mediators ^23, 24^. Indeed, reports show that monocytes are recruited at the site of infections as innate effectors of the inflammatory response to microbes, killing pathogens via phagocytosis, production of reactive oxygen intermediate (ROIs) ^25^, reactive nitrogen intermediate (RNIs) ^26, 27^, myeloperoxidase (MPO) ^28, 29^, and producing inflammatory cytokines ^30^ that contribute to further amplifying the antimicrobial response ^31^.

Human monocytes are derived from hematopoietic stem cells in the bone marrow and are released into peripheral blood circulation upon maturation. They are divided into three major subsets based on the expression of the cell surface markers CD14 and CD16. The most prevalent subset in the blood circulation, accounting for 90% of all monocytes, are the classical monocytes that express high levels of CD14 but low levels of CD16 (CD14++CD16-). The remaining 10% is divided into two subsets: intermediate monocytes with high expression of CD14 and CD16 (CD14++CD16+ or CD14+CD16+) and non-classical monocytes that express low levels of CD14 but high levels of CD16 (CD14dimCD16++ or CD14-CD16++) ^32– 34^. The factors that govern the migration of monocytes and roles that each subset plays during disease processes are not well understood. 1) In autoimmune diseases: Non-classical monocytes are regarded as crucial effectors in the pathogenesis of rheumatoid arthritis, ankylosing spondylitis ^35^, systemic lupus erythematosus (SLE) ^36^ and multiple sclerosis ^37^. This monocyte subset carries a distinct inflammatory signature in patients with SLE ^36^. Classical monocytes on the other hand have been shown to dominate the inflamed mucosa in Crohn’s disease ^38^. Skewing of monocytes towards the intermediate subset has been observed in patients with autoimmune uveitis and linked to administration of glucocorticoid therapy ^39^. 2) In cardiovascular diseases: circulating monocytes play a pivotal role by releasing cocktails of cytokines, factor and proteases that are involved in vascular growth ^40^. Monocyte subsets show functional and phenotypic changes in cardiovascular diseases. The accumulation of classical monocytes is for instance a hallmark of progression of atherosclerosis ^41– 43^. An association between intermediate monocytes and cardiovascular events has also been documented with this monocyte subset being proportionally elevated following myocardial infarction or atrial fibrillation ^44, 45^ or in at risk subjects ^46^. 3) In cancer: Intermediate monocytes are viewed as potential diagnostic indicators for colorectal cancer ^47^. Another study has shown that elevated abundance of intermediate monocytes is associated with survival of adult or childhood acute lymphoblastic leukemia ^48^. The changes of gene expression profiles in monocytes reveal high specificity for the tissue type and cancer histotype, and are induced in response to soluble factors released by the cancer cells in the primary or metastatic site ^49^. Moreover, monocytes, comprising the monocyte-myeloid-derived suppressor cells population, from patients with metastatic breast cancer resemble the reprogrammed immunosuppressive monocytes in patients with severe infections, both by their surface and functional phenotype but also by their gene expression profile ^50^. This signature of immunosuppression could therefore constitute a good biomarker for assessing disease progression. 4) In infections: monocytes are also key players in the immediate immune response to infectious agents as well as the subsequent development of the adaptive immune response ^51^. Given the importance of classical and intermediate monocytes in pathogenesis of infectious and other inflammatory disorders, delineation of their functional and phenotypic characteristics has been studied extensively. The response mounted by classical monocytes has emerged as being critical for the control of a wide range of infectious diseases, including infections caused by bacteria ^52– 57^, parasites ^58^ and fungi ^59^. In contrast, intermediate monocytes have been associated with pathologic immune responses against bacteria ^60, 61^ and parasites ^62^. In the context of HIV infection; CD14 expression is reduced on classical monocytes in chronically HIV-1 infected adults on anti-retroviral therapy ^63, 64^. Moreover, loss of CCR2 expressing non-classical monocytes is associated with cognitive impairment in antiretroviral therapy-naïve infected subjects ^65^. Altogether these findings indicate that monocytes are more than circulating precursors and have different effector functions in response to various infections and during inflammation. Clearly furthering our understanding of the role of monocyte subsets in health and disease will require many more studies, also we hope that the dataset compendium that we are making available to the research community via this publication can help support these endeavors.

In this data note we are making available via GXB a curated compendium of 93 public datasets relevant to human monocyte immunobiology, representing a total of 4,516 transcriptome profiles.

## Materials and methods

### Identification of monocyte datasets

Potentially relevant datasets deposited in GEO were identified using an advanced query based on the Bioconductor package GEOmetadb and the SQLite database that captures detailed information on the GEO data structure; https://www.bioconductor.org/packages/release/bioc/html/GEOmetadb.html ^66^. The search query was designed to retrieve entries where the title and description contained the word Monocyte OR Monocytes, were generated from human samples, using Illumina or Affymetrix commercial platforms. The query result is appended with rich metadata from GEOmetadb that allows for manual filtering of the retrieved collection.

**Figure 1.**
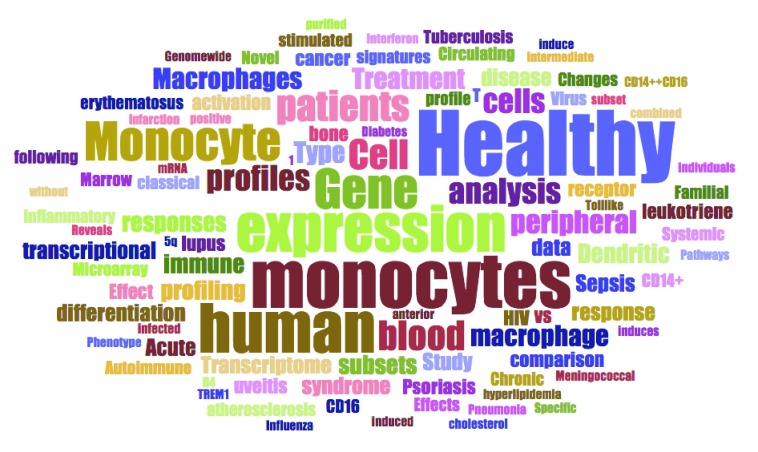
Thematic composition of the dataset collection. Word frequencies extracted from text descriptions of the studies loaded into the GXB tool are depicted as a word cloud. The size of the words is proportional to their frequency.

**Figure 2.**
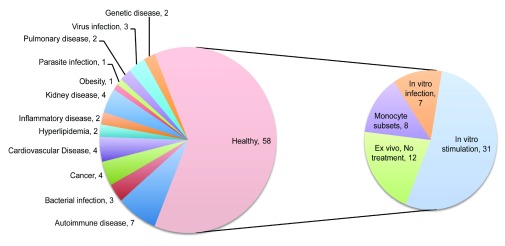
Break down of the dataset collection by category. The pie chart on the left panel indicates dataset frequencies by disease status. The chart on the right panel indicates the type of studies carried out for the 58 datasets consisting of monocyte obtained exclusively from healthy donors.

The relevance of each entry returned by this query was assessed individually. This process involved reading through the descriptions and examining the list of available samples and their annotations. Sometimes it was also necessary to review the original published report in which the design of the study and generation of the dataset is described in more detail. Using the search query, the results also returned a number of datasets that did not include profiles of monocytes but instead of “monocyte-derived dendritic cells” or “monocyte-derived macrophages”. During our manual screen these were excluded as were studies employing monocytic cell lines. Only studies including primary human monocyte profiles were retained. The datasets cover a broad range of studies investigating human monocyte immunobiology in the context of diseases and through comparison with diverse cell populations and study types as illustrated by a graphical representation of relative occurrences of terms in the descriptions of the studies loaded into our tool ( Figure 1). A wide range of cell types and diseases are represented. Ultimately, the collection was comprised of 93 curated datasets. It includes datasets generated from studies profiling primary human CD14+ cells isolated from patients with autoimmune diseases (7), bacterial, virus and parasite infections (7), cancer (4), cardiovascular diseases (4), kidney diseases (4), as well as monocytes isolated from healthy subjects (58) ( Figure 2). The 58 datasets in which monocytes were isolated from healthy subjects were classified based on whether profiling was conducted *ex vivo* or following *in vitro* experiments. In total 38 datasets were identified in which primary human CD14+ cells were stimulated or infected in *in vitro* experiments ( Figure 2). Among the many noteworthy datasets, there are 8 datasets investigating differences between monocytes subsets; classical (CD14++CD16-), intermediate (CD14+CD16+) and non-classical monocytes (CD14-CD16++) ^32– 34^ [GXB: GSE16836, GSE18565, GSE25913, GSE34515, GSE35457, GSE51997, GSE60601, GSE66936]. Another dataset from Banchereau and colleagues investigated responses of monocyte and dendritic cells to 13 different vaccines *in vitro* ^67^ [GXB: GSE44721]. The datasets that comprise our collection are listed in Table 1 and can be browsed interactively in GXB.

**Table 1.** List of datasets constituting the collection.

Title	Platforms	Diseases	Number of samples	Experiments	GEO ID	Ref
Interaction of bone marrow stroma and monocytes: bone marrow stromal cell lines cultured with monocytes	Affymetrix	Healthy	8	*In vitro*	GSE10595	68
Monocyte gene expression profiling in familial combined hyperlipidemia and its modification by atorvastatin treatment	Affymetrix	Familial combined hyperlipidemia	9	*In vitro*	GSE11393	69
Performance comparison of Affymetrix and Illumina microarray technologies	Affymetrix	Acute coronary syndrome	10	*Ex vivo*	GSE11430	70
Gene expression profiling in pediatric meningococcal sepsis reveals dynamic changes in NK-cell and cytotoxic molecules	Affymetrix	Meningococcal sepsis	41	*Ex vivo*	GSE11755	N/A
Effect of interferon-gamma on macrophage differentiation and response to Toll-like receptor ligands	Affymetrix	Healthy	10	*In vitro*	GSE11864	71
Human monocyte and dendritic Cell Subtype Gene Arrays	Affymetrix	Healthy	8	*Ex vivo*	GSE11943	72
Microarray analysis of human monocytes infected with *Francisella tularensis*	Affymetrix	Healthy	14	*In vitro*	GSE12108	73
Human blood monocyte profile in Ventilator-Associated Pneumonia patients	Affymetrix	Pneumonia	60	*Ex vivo*	GSE12838	N/A
Quercetin supplementation and CD14+ monocyte gene expression	Affymetrix	Healthy	6	*Ex vivo*	GSE13899	74
Effects of PMN-Ectosomes on human macrophages	Affymetrix	Healthy	16	*In vitro*	GSE14419	N/A
Homogeneous monocytes and macrophages from hES cells following coculture-free differentiation in M-CSF and IL-3	Affymetrix	Healthy	9	*Ex vivo*	GSE15791	75
Expression data from human macrophages	Affymetrix	Healthy	38	*In vitro*	GSE16385	76
Transcriptional profiling of CD16+ and CD16- peripheral blood monocytes from healthy individuals	Affymetrix	Healthy	8	*Ex vivo*	GSE16836	32
COPD-Specific Gene Expression Signatures of Alveolar Macrophages as well as Peripheral Blood Monocytes Overlap and Correlate with Lung Function	Affymetrix	Chronic Obstructive Pulmonary Disease	12	*Ex vivo*	GSE16972	77
Loss-of-function mutations in REP-1 affect intracellular vesicle transport in fibroblasts and monocytes of CHM patients	Affymetrix	Choroideremia	15	*Ex vivo*	GSE17549	78
Effect of two weeks erythropoietin treatment on monocyte transcriptomes of cardiorenal patients	Illumina	Cardiorenal syndrome	48	*Ex vivo*	GSE17582	N/A
Comparison of gene expression profiles between human monocyte subsets	Affymetrix	Healthy	6	*Ex vivo*	GSE18565	79
Subpopulations of CD163 positive macrophages are classically activated in psoriasis	Illumina	Psoriasis	58	*Ex vivo*	GSE18686	80
Mycobacterium tuberculosis Chaperonin 60.1 has Bipolar Effects on Human peripheral blood-derived Monocytes	Affymetrix	Healthy	21	*In vitro*	GSE18794	N/A
Blood Transcriptional Profiles of Active TB (Separated cell)	Illumina	Tuberculosis	44	*Ex vivo*	GSE19443	11
Filaria induced monocyte dysfunction and its reversal following treatment	Affymetrix	Filariasis	14	*Ex vivo*	GSE2135	81
Ubiquinol-induced gene expression signatures are translated into reduced erythropoiesis and LDL cholesterol levels in humans	Affymetrix	Healthy	6	*Ex vivo*	GSE21351	82
Monocyte vs Macrophage Study	Affymetrix	Healthy	6	*In vitro*	GSE22373	83
Monocyte gene expression patterns distinguish subjects with and without atherosclerosis	Illumina	Carotid atherosclerosis	95	*Ex vivo*	GSE23746	N/A
Deconvoluting Early Post-Transplant Immunity Using Purified Cell Subsets Reveals Functional Networks Not Evident by Whole Blood Analysis	Affymetrix	Kidney Transplantation	179	*Ex vivo*	GSE24223	84
Cooperative and redundant signaling of leukotriene B4 and leukotriene D4 in human monocytes	Affymetrix	Healthy	10	*In vitro*	GSE24869	85
Gene expression profiling of the classical (CD14++CD16-), intermediate (CD14++CD16+) and nonclassical (CD14+CD16+) human monocyte subsets	Illumina	Healthy	24	*Ex vivo*	GSE25913	34
Direct Cell Conversion of Human Fibroblasts to Monocytic phagocytes by Forced Expression of Monocytic Regulatory Network Elements	Illumina	Dermatomyositis	15	*Ex vivo*	GSE27304	N/A
cMyb and vMyb in human monocytes	Affymetrix	Healthy	6	*In vitro*	GSE2816	86
Temporal transcriptional changes in human monocytes following acute myocardial infarction: The GerMIFs monocyte expression study	Illumina	Acute myocardial infarction	76	*Ex vivo*	GSE28454	N/A
mRNA expression profiling of human immune cell subset (Roche)	Affymetrix	Healthy	47	*Ex vivo*	GSE28490	87
mRNA expression profiling of human immune cell subsets (HUG)	Affymetrix	Healthy	33	*Ex vivo*	GSE28491	87
Changes in gene expression profiles in patients with 5q- syndrome in CD14+ monocytes caused by lenalidomide treatment	Illumina	5q- syndrome	17	*Ex vivo*	GSE31460	N/A
Genome-wide analysis of lupus immune complex stimulation of purified CD14+ monocytes and how this response is regulated by C1q	Illumina	Healthy	8	*In vitro*	GSE32278	88
Transcriptome analysis of circulating monocytes in obese patients before and three months after bariatric surgery	Illumina	Obesity	48	*Ex vivo*	GSE32575	89
CD4 on human monocytes	Affymetrix	Healthy	6	*In vitro*	GSE32939	90
Peripheral Blood Monocyte Gene Expression in Recent-Onset Type 1 Diabetes	Illumina	Type 1 Diabetes	22	*Ex vivo*	GSE33440	91
Traffic-related Particulate Matter Upregulates Allergic Responses by a Notch-pathway Dependent Mechanism	Affymetrix	Healthy	16	*In vitro*	GSE34025	N/A
Human monocyte activation with NOD2L vs. TLR2/1L	Affymetrix	Healthy	45	*In vitro*	GSE34156	92
Bacillus anthracis' lethal toxin induces broad transcriptional responses in human peripheral monocyte	Affymetrix	Healthy	8	*In vitro*	GSE34407	93
Gene expression profiles of human blood classical monocytes (CD14++CD16-), CD16 positive monocytes (CD14+16++ and CD14++CD16+), and CD1c+ CD19- dendritic cells	Affymetrix	Healthy	9	*Ex vivo*	GSE34515	N/A
Genome-wide analysis of monocytes and T cells' response to interferon beta	Illumina	Healthy	12	*In vitro*	GSE34627	94
Highly pathogenic influenza virus inhibit Inflammatory Responses in Monocytes via Activation of the Rar-Related Orphan Receptor Alpha (RORalpa)	Affymetrix	Healthy	12	*In vitro*	GSE35283	N/A
Transcriptome profiles of human monocyte and dendritic cell subsets	Illumina	Healthy	49	*Ex vivo*	GSE35457	95
Influenza virus A infected monocytes	Illumina	Healthy	6	*In vitro*	GSE35473	96
PGE2-induced OSM expression	Affymetrix	Chronic wound	6	*Ex vivo*	GSE36995	97
Inflammatory Expression Profiles in Monocyte to Macrophage Differentiation amongst Patients with Systemic Lupus Erythematosus and Healthy Controls with and without an Atherosclerosis Phenotype	Illumina	Systemic lupus erythematosus	72	*Ex vivo*	GSE37356	N/A
New insights into key genes and pathways involved in the pathogenesis of HLA-B27-associated acute anterior uveitis	Affymetrix	Acute anterior uveitis	6	*In vitro*	GSE37588	N/A
Analysis of blood myelomonocytic cells from RCC patients	Illumina	Renal cell carcinoma	8	*Ex vivo*	GSE38424	98
Nanotoxicogenomic study of ZnO and TiO2 responses	Illumina	Healthy	90	*In vitro*	GSE39316	N/A
Macrophage Microvesicles Induce Macrophage Differentiation and miR-223 Transfer	Affymetrix	Healthy	24	*In vitro*	GSE41889	99
TREM-1 is a novel therapeutic target in Psoriasis	Affymetrix	Psoriasis	15	*In vitro*	GSE42305	100
Comparison study between Uremic patient with Healthy control	Affymetrix	Chronic kidney disease	6	*Ex vivo*	GSE43484	N/A
Microarray analysis of IL-10 stimulated adherent peripheral blood mononuclear cells	Affymetrix	Healthy	8	*In vitro*	GSE43700	101
Monocytes and Dendritic cells stimulated by 13 human vaccines and LPS	Illumina	Vaccination	128	*In vitro*	GSE44721	67
Gene expression profile of human monocytes stimulated with all-trans retinoic acid (ATRA) or 1,25a-dihydroxyvitamin D3 (1,25D3)	Affymetrix	Healthy	12	*In vitro*	GSE46268	102
Transcriptome analysis of blood monocytes from sepsis patients	Illumina	Sepsis	44	*Ex vivo*	GSE46955	103
Tumor-educated circulating monocytes are powerful specific biomarkers for diagnosis of colorectal cancer	Illumina	Colorectal Cancer	93	*Ex vivo*	GSE47756	49
Similarities and differences between macrophage polarized gene profiles	Illumina	Healthy	12	*In vitro*	GSE49240	104
The effect of cell subset isolation method on gene expression in leukocytes.	Illumina	Healthy	50	*Ex vivo*	GSE50008	N/A
Transcriptome analysis of HIV-infected peripheral blood monocytes	Illumina	HIV	86	*Ex vivo*	GSE50011	105
Gene expression profiles in T-lymphocytes and Monocytes of participants of the Tour de France 2005	Affymetrix	Healthy	66	*Ex vivo*	GSE5105	N/A
Effects of exercise on gene expression level in human monocytes	Affymetrix	Healthy	24	*Ex vivo*	GSE51835	106
T helper lymphocyte- and monocyte-specific type I interferon (IFN) signatures in autoimmunity and viral infection.	Affymetrix	Autoimmune diseases	36	*Ex vivo*	GSE51997	107
Longitudinal comparison of monocytes from an HIV viremic vs avirmeic state	Affymetrix	HIV	16	*Ex vivo*	GSE5220	108
Expression data from monocytes and monocyte derived macrophages	Affymetrix	Healthy	12	*In vitro*	GSE52647	N/A
Transcriptome analysis of primary monocytes from HIV+ patients with differential responses to therapy	Illumina	HIV	14	*Ex vivo*	GSE52900	109
Human blood monocyte response to IL-17A in culture	Affymetrix	Healthy	6	*In vitro*	GSE54884	N/A
Divergent genome wide transcriptional profiles from immune cell subsets isolated from SLE patients with different ancestral backgrounds	Illumina	Systemic lupus erythematosus	208	*Ex vivo*	GSE55447	110
Cell Specific Expression & Pathway Analyses Reveal Novel Alterations in Trauma-Related Human T-Cell & Monocyte Pathways	Affymetrix	Trauma patients	42	*Ex vivo*	GSE5580	111
Immune Variation Project (ImmVar) [CD14]	Affymetrix	Healthy	485	*Ex vivo*	GSE56034	N/A
Transcriptomics of human monocytes	Illumina	Healthy	1202	*Ex vivo*	GSE56045	112
Effect of vitamin D treatment on human monocyte	Affymetrix	Healthy	16	*In vitro*	GSE56490	NA
Monocytes of patients with familial hypercholesterolemia show alterations in cholesterol metabolism	Affymetrix	Hypercholesterolemia	23	*Ex vivo*	GSE6054	113
Gene expression data from CD14++ CD16- classical monocytes from healthy volunteers and patients with pancreatic ductal adenocarcinoma	Affymetrix	Pancreatic ductal adenocarcinoma	12	*Ex vivo*	GSE60601	N/A
Activation of the JAK/STAT pathway in Behcet's Disease	Affymetrix	Behcet’s Disease	29	*Ex vivo*	GSE61399	N/A
Alarmins MRP8 and MRP14 induce stress-tolerance in phagocytes under sterile inflammatory conditions	Illumina	Sterile Inflammation	12	*In vitro*	GSE61477	N/A
GM-CSF induced gene-regulation in human monocytes	Affymetrix	Healthy	6	*In vitro*	GSE63662	114
Treatment of human monocytes with TLR7 or TLR8 agonists	Affymetrix	Healthy	9	*In vitro*	GSE64480	115
Restricted Dendritic Cell and Monocyte Progenitors in Human Cord Blood and Bone Marrow	Illumina	Healthy	36	*Ex vivo*	GSE65128	116
Interleukin-1- and Type I Interferon-Dependent Enhanced Immunogenicity of an NYVAC-HIV-1 Env-Gag-Pol-Nef Vaccine Vector with Dual Deletions of Type I and Type II Interferon-Binding Proteins	Illumina	Vaccination	20	*In vitro*	GSE65412	NA
Comparative analysis of monocytes from healthy donors, patients with metastatic breast cancer, sepsis or tuberculosis.	Illumina	Breast cancer and Bacterial infection	13	*Ex vivo*	GSE65517	50
Expression data from intermediate monocytes from healthy donors and autoimmune uveitis patients	Affymetrix	Autoimmune uveitis	21	*Ex vivo*	GSE66936	39
Induction of Dendritic Cell-like Phenotype in Macrophages during Foam Cell Formation	Affymetrix	Healthy	22	*In vitro*	GSE7138	117
Genome Wide Gene Expression Study of Circulating Monocytes in human with extremely high vs. low bone mass	Affymetrix	Healthy	26	*Ex vivo*	GSE7158	N/A
Genomic profiles for human peripheral blood T cells, B cells, natural killer cells, monocytes, and polymorphonuclear cells: comparisons to ischemic stroke, migraine, and Tourette syndrome	Affymetrix	Healthy	18	*Ex vivo*	GSE72642	118
Expression data from monocytes of individuals with different collateral flow index CFI	Affymetrix	Coronary artery disease	160	*Ex vivo*	GSE7638	39
Leukotriene D4 induces gene expression in human monocytes through cysteinyl leukotriene type I receptor	Affymetrix	Healthy	8	*In vitro*	GSE7807	119
Gene expression profile during monocytes to macrophage differentiation	Affymetrix	Healthy	9	*In vitro*	GSE8286	N/A
Toll-like receptor triggering of a vitamin D-mediated human antimicrobial response	Affymetrix	Healthy	50	*In vitro*	GSE8921	120
TRAIL Is a Novel Antiviral Protein against Dengue Virus	Affymetrix	Dengue	10	*In vitro*	GSE9378	NA
Gene Expression-Based High Throughput Screening: APL Treatment with Candidate Compounds	Affymetrix	Leukemia	24	*Ex vivo*	GSE976	121
Innate immune responses to TREM-1 activation	Affymetrix	Healthy	11	*In vitro*	GSE9988	122

### Dataset upload and annotation on GXB

Once a final selection was made each dataset was downloaded from GEO in the SOFT file format. It was in turn uploaded on an instance of the Gene Expression Browser (GXB) hosted on the Amazon Web Services cloud. Available sample and study information were also uploaded. Samples were grouped according to possible interpretations of study results and ranking based on the different group comparisons that were computed (e.g. comparing monocyte isolated from case vs controls in studies where profiling was performed *ex-vivo*; or stimulated vs medium control in *in vitro* experiments).

### Short Gene Expression Brower tutorial

The GXB software has been described in detail in a recent publication ^20^. This custom software interface provides users with a means to easily navigate and filter the dataset collection available at http://monocyte.gxbsidra.org/dm3/landing.gsp. A web tutorial is also available online: http://monocyte.gxbsidra.org/dm3/tutorials.gsp#gxbtut. Briefly, datasets of interest can be quickly identified either by filtering using criteria from pre-defined lists on the left or by entering a query term in the search box at the top of the dataset navigation page. Clicking on one of the studies listed in the dataset navigation page opens a viewer designed to provide interactive browsing and graphic representations of large-scale data in an interpretable format. This interface is designed to present ranked gene lists and display expression results graphically in a context-rich environment. Selecting a gene from the rank ordered list on the left of the data-viewing interface will display its expression values graphically in the screen’s central panel. Directly above the graphical display drop down menus give users the ability: a) To change how the gene list is ranked; this allows the user to change the method used to rank the genes, or to include only genes that are selected for specific biological interest; b) To change sample grouping (Group Set button), in some datasets a user can switch between groups based on cell type to groups based on disease type, for example; c) To sort individual samples within a group based on associated categorical or continuous variables (e.g. gender or age); d) To toggle between the bar chart view and a box plot view, with expression values represented as a single point for each sample. Samples are split into the same groups whether displayed as a bar chart or box plot; e) To provide a color legend for the sample groups; f) To select categorical information that is to be overlaid at the bottom of the graph. For example, the user can display gender or treatment status in this manner; g) To provide a color legend for the categorical information overlaid at the bottom of the graph; and h) To download the graph as a png image or csv file for performing a separate analysis. Measurements have no intrinsic utility in absence of contextual information. It is this contextual information that makes the results of a study or experiment interpretable. It is therefore important to capture, integrate and display information that will give users the ability to interpret data and gain new insights from it. We have organized this information under different tabs directly above the graphical display. The tabs can be hidden to make more room for displaying the data plots, or revealed by clicking on the blue “show info panel” button on the top right corner of the display. Information about the gene selected from the list on the left side of the display is available under the “Gene” tab. Information about the study is available under the “Study” tab. Information available about individual samples is provided under the “Sample” tab. Rolling the mouse cursor over a bar chart's element while displaying the “Sample” tab lists any clinical, demographic, or laboratory information available for the selected sample. Finally, the “Downloads” tab allows advanced users to retrieve the original dataset for analysis outside this tool. It also provides all available sample annotation data for use alongside the expression data in third party analysis software. Other functionalities are provided under the “Tools” drop-down menu located in the top right corner of the user interface. Some of the notable functionalities available through this menu include: a) Annotations, which provides access to all the ancillary information about the study, samples and dataset organized across different tabs; b) Cross-project view, which provides the ability for a given gene to browse through all available studies; c) Copy link, which generates a mini-URL encapsulating information about the display settings in use and that can be saved and shared with others (clicking on the envelope icon on the toolbar inserts the url in an email message via the local email client); and d) Chart options, which gives user the option to customize chart labels.

### Dataset validation

**Figure 3.**
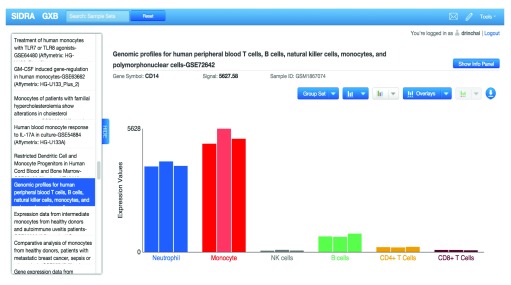
Illustrative example showing the abundance levels of CD14 transcripts across samples in a given study. The expression of this gene is indicative of the purity of primary human monocyte preparation; this marker is expected to be high in monocyte preparations and low in other leukocyte populations. In this view of the GXB expression of CD14 can be visualized across projects listed on the left.

Quality control checks were performed with the examination of profiles of relevant biological indicators. Known leukocyte markers were used, such as CD14, which is expressed by monocytes and macrophages; as well as markers that would indicate significant contamination of the sample by other leukocyte populations: such as CD3, a T-cells marker; CD19, a B-cell marker; CD56, an NK cell marker ( Figure 3; The expression of the CD14 marker across all studies can be checked using the cross project functionality of GXB: http://monocyte.gxbsidra.org/dm3/geneBrowser/crossProject?probeID=201743_at&geneSymbol=CD14&geneID=929). We have systematically verified that expression of the genes encoding those surface markers was consistent with grouping labels provided by depositors. In addition, expression of the XIST transcripts, in which expression is gender-specific, was also examined to determine its concordance with demographic information provided with the GEO submission (expression of XIST should be high in females and low in males).

## Data availability

The data referenced by this article are under copyright with the following copyright statement: Copyright: © 2016 Rinchai D et al.

Data associated with the article are available under the terms of the Creative Commons Zero "No rights reserved" data waiver (CC0 1.0 Public domain dedication). 

All datasets included in our curated collection are also available publically via the NCBI GEO website: http://www.ncbi.nlm.nih.gov/geo/; and are referenced throughout the manuscript by their GEO accession numbers (e.g. GSE25913). Signal files and sample description files can also be downloaded from the GXB tool under the “downloads” tab.
