# Supplementary material for: A curated compendium of monocyte transcriptome datasets of relevance to human monocyte immunobiology research
Source: F1000Res. 2016 Apr 25;5:291. Originally published 2016 Mar 7. [Version 2] doi: 10.12688/f1000research.8182.2 (PMC4856112; doi:10.12688/f1000research.8182.2)
Supplement: Peer review history

# Peer review: Referee response for version 2

**DOI:** 10.5256/f1000research.9108.r13544

**Published:** 2016-05-03

**Authors:** Ping Chen, David Kuo

**Affiliations:** 1 National Eye Institute (NEI), National Institutes of Health, Besthesda, MD, USA; 2 National Eye Institute (NEI), National Institutes of Health, Bethesda, MD, USA; 3 University of California San Diego, La Jolla, CA, USA

The authors have addressed the concerns appropriately.

We have read this submission. We believe that we have an appropriate level of expertise to confirm that it is of an acceptable scientific standard.

# Peer review: Referee response for version 1

**DOI:** 10.5256/f1000research.8800.r12769

**Published:** 2016-03-21

**Authors:** Ping Chen, David Kuo

**Affiliations:** 1 National Eye Institute (NEI), National Institutes of Health, Besthesda, MD, USA; 2 National Eye Institute (NEI), National Institutes of Health, Bethesda, MD, USA; 3 University of California San Diego, La Jolla, CA, USA

**General Comments**

Modern genomics, especially with the emergence of high-throughput next-generation sequencing, is generating data at such a rapid rate that new tools for organizing, visualizing, sharing, and integrating heterogeneous data in the context of scientific information are needed for scientists to efficiently use these published data. The Chaussabel group has recently developed an interactive data browsing and visualization web application, the Gene Expression Browser (GXB), to address this problem.

In this data note, Dr. Rinchai *et al.* report a compendium of ninety-six curated human monocyte transcriptome datasets from GEO spanning a broad range of diseases, cell types, and experiments. These datasets were then uploaded to the Gene Expression Browser for exploratory data analysis and dataset validation. The Gene Expression Browser should prove very useful for investigating large datasets; however, I have several questions and comments regarding the curated data itself:

**Title:**

The novel aspect and apparent emphasis of this data note is using the Gene Expression Browser to more easily explore the curated ninety-six datasets. But the current title emphasizes the key information on fostering the knowledge discovery. Please consider rephrasing it by focusing on the monocyte datasets and web application.

**Introduction:**

As the Gene Expression Browser has been described in detail previously, the emphasis of this data note should be on the curated data. It would be helpful to discuss the motivation for creating this particular compendium of monocyte transcriptome datasets as well as the intended use of the curated data given the breadth and heterogeneity of diseases, cell types, and experiments that it includes.

**Methods:**

1. Please elaborate more specifically on how the datasets were curated. What were the eligibility criteria for inclusion into the compendium?

2. The table summarizing the published data can difficult to read due to its landscape orientation. Consider rotating the table from a landscape orientation to a portrait orientation.

3. In the right pie chart of Figure 2, there are twelve datasets studying primary monocytes; however, datasets classified as *in vitro* stimulation, infection, and monocyte subsets may also contain primary monocytes. Better categorization is needed.

4. Data validation is critical for verifying that a dataset is acceptable for use.  The authors mention performing dataset validation but do not report the related results or summary of their validation.  On page 9, the process of assessing contamination by other leukocyte populations using surface markers should be done carefully as CD14 ^+^ monocytes do share surface marker CD4.

5. In Fig. 3, it is unclear whether the orange bar plot is referring to CD4 ^+^ T cells or CD4 ^+^ cells in general. They are different cell types.

We have read this submission. We believe that we have an appropriate level of expertise to confirm that it is of an acceptable scientific standard, however we have significant reservations, as outlined above.

# Response

**Published:** 2016-03-29

**Authors:** Darawan Rinchai

**Affiliations:** Sidra Medical and Research Center, Qatar

We thank the reviewers for their valuable feedback and suggestions to improve our manuscript.

**Title:**

*Following the suggestion of the reviewers we changed the title of the manuscript to “A curated compendium of of transcriptome datasets of relevance to human monocyte immunobiology research”.*

**Introduction:**

*Thanks for raising this point. We added a long paragraph and new references in the introduction to emphasize the role of monocyte across different diseases and the motivation for creating this compendium of monocyte transcriptome datasets.*

**Methods:**

1. *We have added information about how datasest were selected for inclusion in the collections in the methods section under the title “Identification of monocyte datasets”… **“*** *Using the search query, the results also returned a number of datasets that did not include profiles of monocytes but instead of “monocyte-derived dendritic cells” or “monocyte-derived macrophages”. During our manual screen these were excluded as were studies employing monocytic cell lines. Only studies including primary human monocyte profiles were retained.”…*

2. *We agree with the reviewer that presenting the table using landscape orientation makes it difficult to read. We therefore changed table format from landscape to portrait orientation.*

3. *Thank you for pointing this out. We changed the label on this figure to read “ex-vivo, no treatment”. These include studies where monocytes were isolated from healthy subjects for comparison with other cell types, or evaluation of variation among healthy individuals.*

4. *Assessing contamination can indeed be difficult, especially using this type of data where cell-level information is lacking. We plan to explore with our bioinformatics collaborators the development of a "scoring" approach to better quantify potential contamination but this is not a simple matter to address. At this point we have simply verified for each dataset that expression of markers was consistent with grouping labels provided by depositors. We have added language in the manuscript to clarify this point.*

5. *Thank you for pointing out this typo on this label. This dataset focuses on genomic profile of human blood both CD4+ and CD8+ T cells, B cells, NK cells monocytes and neutrophil. Figure 3 was corrected accordingly as shown in the new Figure 3.*

# Peer review: Referee response for version 1

**DOI:** 10.5256/f1000research.8800.r12768

**Published:** 2016-03-16

**Authors:** Marc Pellegrini

**Affiliations:** 1 Division of Infection and Immunity, Walter and Eliza Hall Institute of Medical Research, Parkville, VIC, Australia

In this short descriptive report the authors put their published Gene Expression Browser tool to work in arranging several thousand transcriptome profiles obtained from public datasets that looked at monocyte immunology. They were able to compare groups of monocytes based on phenotypic attributes and rank gene expression. The authors provide a nice summary of the technique and validation.

I have read this submission. I believe that I have an appropriate level of expertise to confirm that it is of an acceptable scientific standard.
